# Supplementary material for: Engineering inter-promoter spacing in baculovirus dual-expression systems enhances transcription and reduces rAAV2 empty capsids
Source: Mol Ther Adv. 2026 Jun 23;34(3):201792. doi: 10.1016/j.omta.2026.201792 (PMC13355693; doi:10.1016/j.omta.2026.201792)
Supplement: Document S1. Figure S1 and Table S1 [file mmc1.pdf]

OMTA, Volume *34*

Supplemental information

Engineering inter-promoter spacing in baculovirus dual-expression systems enhances transcription and reduces rAAV2 empty capsids

Lan Lan, Yuchen Qu, Jie Wang, Wentao Wu, Yao Chen, Weijin Meng, Wangcheng Song, Yongqiang Hou, Lijun Shi, Hao Nan, and Xiaodong Xu

Table S1. Primers used in this study.

Primers	Sequence (5′-3′)
mSc-F	ttacttgtcatcgtcgtccttgtaatcggatcccttgtacagctcgtccatgccgccgg
mSc-R	atggtgagcaagggcgaggc
EGFP-F	atgagcaagggcgaggagct
EGFP-R	ttagtggtggtggtggtggtgctcgagcttgtacagctcgtccatgccgagagtgatcc
RFP-F	atggcggaaggctccgtcgc
RFP-R	gtggtggtggtggtggtgctcgaggctctcaagcgcggtgatccg
phRFP-R	gacggagccttccgccatatttataggtttttttattacaaaactg
p10-F	cgcccttgctcaccataagcttgattgtaaataaaatgtaatttacag
polh-R	cctcgcccttgctcatgaattcatttataggtttttttattacaaaactg
105-F	cgtatactccggaatattaatagatcatggagataattaaaatg
105-R	ttaatattccggagtatacggacctttaattcaacccaac
119-F	tcactcccaatagctgtcatg
119-R	atgacagctattgggagtgagacctttaattcaacccaac
133-F	caatagctgtcatggacgccatattaatagatcatggagataattaaaatgataaccat
133-R	ggcgtccatgacagctattgggagtgacagtaacggctagcgacctttaattcaacc
147-F	ttgatgactacgcagctagccgttactgtcactc
147-R	gctagctgcgtagtcatcaagacctttaattcaacccaac
161-F	ttgatgactacgcaacataaccggatacgctagccgttactgtcactc
161-R	gtatccggttatgttgcgtagtcatcaagacctttaattcaacccaac
175-F	tacgcaaccgacagacttggacataaccggatacgctagc
175-R	ccaagtctgtcggttgcgtagtcatcaagacctttaattc
189-F	accgacagacttggactgaggccagcgtacataaccggatacgctagc
189-R	acgctggcctcagtccaagtctgtcggttgcgtagtcatcaagacctttaattc
203-F	gcgtcctcttcctgaaggacataaccggatacgctagc
203-R	atgtccttcaggaagaggacgctggcctcagtccaagt
217-F	cttcctgaaggccagaatccacaccacataaccggatacgctagc
217-R	gtggattctggccttcaggaagaggacgctggcctcagtccaagt
231-F	aaggccagaatccacacccctatcctgcgcaaacataaccggatacgctagc
231-R	taggggtgtggattctggccttcaggaagaggacgctggcctcagtccaagt
245-F	gaatccacacccctatcctgcgcaatcaagctgagacaaacataaccggatacgctagc
245-R	cgcaggataggggtgtggattctggccttcaggaagaggacgctggcctcagtccaagt
141-F	aaggtcactacgcagctagccgttactgtcactc
141-R	gctagctgcgtagtgacctttaattcaacccaacac
143-F	ggtctgactacgcagctagccgttactgtcactc
143-R	gctagctgcgtagtcagacctttaattcaacccaacac
145-F	ggtcgatgactacgcagctagccgttactgtcac
145-R	tagctgcgtagtcatcgacctttaattcaacccaacac

149-F	gatgactacgcaacgctagccgttactgtcactc
149-R	gctagcgttgcgtagtcatcaagacctttaattc
151-F	tgactacgcaacatgctagccgttactgtcactc
151-R	gctagcatgttgcgtagtcatcaagacctttaattc
153-F	tgactacgcaacataagctagccgttactgtcactc
153-R	gctagcttatgttgcgtagtcatcaagacctttaattc
155-F	gactacgcaacataaccgctagccgttactgtcactc
155-R	ctagcggttatgttgcgtagtcatcaagacctttaattc
157-F	gactacgcaacataaccgggctagccgttactgtcactc
157-R	cccggttatgttgcgtagtcatcaagacctttaattc
159-F	actacgcaacataaccggatgctagccgttactgtcactc
159-R	atccggttatgttgcgtagtcatcaagacctttaattc
R140-F	ttccgcaatactattctaattcttcacatcgcattgaattcatcatggagataattaaaatgataac
R140-R	attagaatagtattgcggaataacacgagcgaagcttgacctttaattcaacccaacac
R141-F	ggttgaattaaaggtcaagcttcgctcgtgttattccgcaatactattctaattcttca
R141-R	taattatctccatgatgaattcgaatgcgatgtgaagaattagaatagtattgcgg
R142-R	taattatctccatgatgaattccgaatgcgatgtgaagaattagaatagtattgcgg
R143-R	taattatctccatgatgaattctcgaatgcgatgtgaagaattagaatagtattgcgg
R144-R	taattatctccatgatgaattcgtcgaatgcgatgtgaagaattagaatagtattgcgg
R145-R	taattatctccatgatgaattctgtcgaatgcgatgtgaagaattagaatagtattgcgg
R146-R	taattatctccatgatgaattcatgtcgaatgcgatgtgaagaattagaatagtattgcgg
R147-R	taattatctccatgatgaattcgatgtcgaatgcgatgtgaagaattagaatagtattgcgg
R148-R	taattatctccatgatgaattcagatgtcgaatgcgatgtgaagaattagaatagtattgcgg
R149-R	taattatctccatgatgaattctagatgtcgaatgcgatgtgaagaattagaatagtattgcgg
R150-R	taattatctccatgatgaattcttagatgtcgaatgcgatgtgaagaattagaatagtattgcgg
R151-R	taattatctccatgatgaattcgttagatgtcgaatgcgatgtgaagaattagaatagtattgcgg
R152-R	taattatctccatgatgaattccgttagatgtcgaatgcgatgtgaagaattagaatagtattgcgg
R153-R	taattatctccatgatgaattctcgttagatgtcgaatgcgatgtgaagaattagaatagtattgcgg
R154-R	taattatctccatgatgaattcgtcgttagatgtcgaatgcgatgtgaagaattagaatagtattgcgg
R155-R	taattatctccatgatgaattcagtcgttagatgtcgaatgcgatgtgaagaattagaatagtattgcgg
R156-R	taattatctccatgatgaattcgagtcgttagatgtcgaatgcgatgtgaagaattagaatagtattgcgg
R157-R	taattatctccatgatgaattctgagtcgttagatgtcgaatgcgatgtgaagaattagaatagtattgcgg
R158-R	taattatctccatgatgaattcatgagtcgttagatgtcgaatgcgatgtgaagaattagaatagtattgcgg
R159-R	taattatctccatgatgaattcaatgagtcgttagatgtcgaatgcgatgtgaagaattagaatagtattgcgg
R160-R	taattatctccatgatgaattcgaatgagtcgttagatgtcgaatgcgatgtgaagaattagaatagtattgcgg

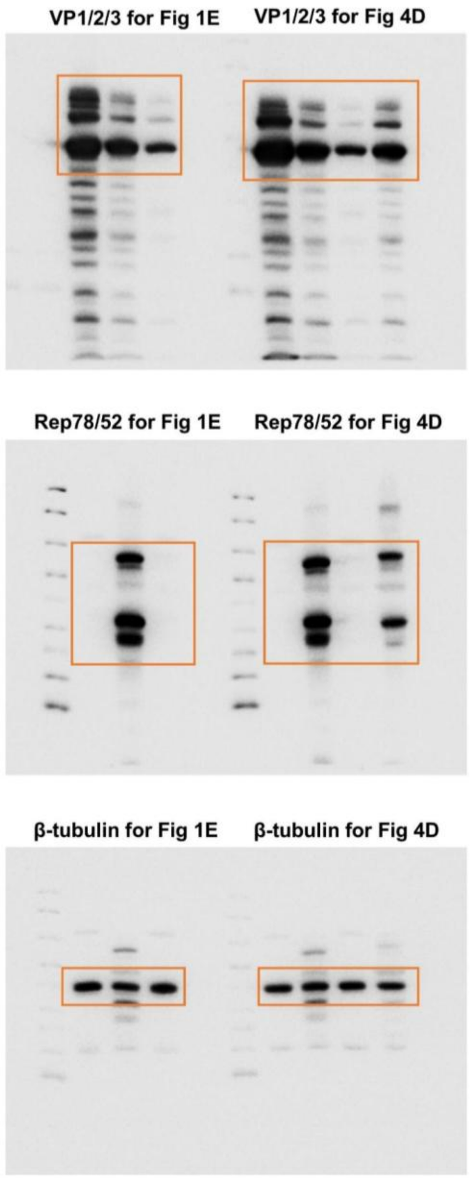

Figure S1. Uncropped scans of Western blots used in Figures 1 and 4.

Original scans of the Western blot analysis. This figure displays the original, unedited blot membranes corresponding to the cropped images presented in the main text. Top panels show the blots probed for VP1/2/3, middle panels for Rep78/52, and bottom panels for the loading control β-tubulin. The specific areas enclosed by the orange rectangles denote the cropped sections shown in Figure 1E and Figure 4D.
