# Supplementary material for: Engineering inter-promoter spacing in baculovirus dual-expression systems enhances transcription and reduces rAAV2 empty capsids
Source: Mol Ther Adv. 2026 Jun 23;34(3):201792. doi: 10.1016/j.omta.2026.201792 (PMC13355693; doi:10.1016/j.omta.2026.201792)
Supplement: Document S2. Article plus supplemental information [file mmc2.pdf]

Molecular Therapy
Advances
Original Article

Engineering inter-promoter spacing in baculovirus dual-expression systems enhances transcription and reduces rAAV2 empty capsids

Lan Lan,[1] Yuchen Qu,[1] Jie Wang,[1] Wentao Wu,[1] Yao Chen,[2] Weijin Meng,[2] Wangcheng Song,[1] Yongqiang Hou,[3] Lijun Shi,[2] Hao Nan,[1] and Xiaodong Xu[1]

[1]College of Life Sciences, Northwest A&F University, Yangling 712100, Shaanxi, China; [2]R&D Department, Xi'an uniBac Biotechnology Co., Ltd., Xi'an 710000, Shaanxi, China; [3]R&D Department, Shaanxi Bacmid Biotechnology Co., Ltd., Yangling 712100, Shaanxi, China

Global delivery of gene therapies demands scalable manufacturing, yet the field is limited by the genome-deficient empty capsids. While the baculovirus expression vector system (BEVS) serves as a leading platform for industrial scale-up, its packaging efficiency is frequently compromised. This failure is largely driven by an imbalanced expression of Rep and Cap proteins. Here, we showed that the conventional back-to-back arrangement of *p10* and *polh* promoters induced transcriptional interference, which suppressed viral protein stoichiometry and limited genome encapsulation. Using systematic dual-fluorescence reporter system, we discover an unexpected non-linear response, in which specific spacer lengths (133–161 bp) paradoxically enhance bidirectional transcription rather than simply relieving transcription repression. Nucleotide-resolution mapping identified a sharp structural optimum at 153 bp that triggered transcriptional enhancement beyond separated promoter controls. Integrating this 153 bp configuration into functional rAAV2 packaging systems restored Rep and Cap expression. Consequently, genome replication efficiency tripled, and the accumulation of empty capsids dropped precipitously from 84.9% to 20.1%, while transduction efficiency in mammalian cells remained uncompromised. These findings define a design rule for dual-promoter expression vector that is readily implementable in rAAV manufacturing. This work establishes a translatable engineering strategy to enhance both product quality and manufacturing efficiency for rAAV-based gene therapies.

INTRODUCTION

Gene therapy has emerged as a promising treatment for a variety of inherited and complex diseases.[1] Recombinant adeno-associated virus (rAAV) has been established as the leading platform for *in vivo* gene therapy due to its safety profile, low immunogenicity, and capacity for sustained transgene expression.[2,3] With numerous drug approvals and an expanding clinical pipeline, rAAV manufacturing is transitioning from laboratory development to large scale industrialization, where single batch requirements have reached the range of 10^{14} to 10^{16} vector genomes (vg). However, the gap between production capacity and product consistency remains a significant barrier to global accessibility.[4] The generation of empty particles, which are nonfunctional particles lacking the therapeutic genome, is a primary challenge for the industry.[5,6] These empty particles not only dilute therapeutic potency but also trigger undesirable immune responses and increase the complexity of downstream purification.[7,8] Minimizing the empty particles fraction while maintaining high yields has become a critical engineering benchmark for evaluating AAV production processes.[9]

The baculovirus expression vector system (BEVS) is a scalable platform for rAAV production due to its capacity for high-density suspension culture and linear scalability.[10] However, BEVS frequently suffers from compromised packaging efficiency compared to mammalian cell systems, with empty capsid rates often exceeding 90%.[11] This quality bottleneck results from an imbalanced expression of Rep proteins (responsible for genome replication and packaging) and Cap proteins (forming the viral capsid).[12] In BEVS, where multiple transgenes must be coordinated, insufficient Rep expression or temporal mismatch between genome replication and capsid assembly might lead to empty capsid accumulation.[13,14] Therefore, restoring the precise balance of Rep and Cap expression represents a fundamental strategy to overcome the intrinsic quality limitations in BEVS-based rAAV production.

Prior optimization efforts for BEVS-based rAAV production have focused on host cell and culture conditions, multiplicity of infection (MOI) adjustment, codon and untranslated region (UTR)

Received 15 January 2026; accepted 17 June 2026;
https://doi.org/10.1016/j.omta.2026.201792.

Correspondence: Lijun Shi, R&D Department, Xi'an uniBac Biotechnology Co., Ltd., Xi'an 710000, Shaanxi, China.
E-mail: helloshilijun@163.com
Correspondence: Hao Nan, College of Life Sciences, Northwest A&F University, Yangling 712100, Shaanxi, China.
E-mail: nanhao@nwsuaf.edu.cn
Correspondence: Xiaodong.Xu, College of Life Sciences, Northwest A&F University, Yangling 712100, Shaanxi, China.
E-mail: xuxd@nwsuaf.edu.cn

© 2026 The Author(s).
Published by Elsevier Inc. on behalf of The American Society of Gene and Cell Therapy.
This is an open access article under the CC BY license (http://creativecommons.org/licenses/by/4.0/).

engineering, and protein modifications.[15–18] These interventions have improved total rAAV titers or reduced empty particle rates, but systematic investigation of structural parameters within back-to-back bidirectional promoter cassettes remains limited. Specifically, the bidirectional promoter configuration (typically utilizing the *p10* and *polh* promoters) used to co-express Rep and Cap is largely a legacy design inherited from early baculovirus systems intended for multi-protein complex production.[19,20] In classical BEVS, the primary focus was maximizing total protein yield, with little attention paid to how geometric arrangement between promoters might fine-tune transcriptional efficiency.[21,22] This historical constraint has resulted in largely empirical, fixed spacing between *p10* and *polh* promoters in current rAAV production vectors.[23] The potential of this physical distance as an adjustable engineering parameter to modulate Rep/Cap ratios and reduce empty capsid rates has not been systematically evaluated.

The closed arrangement of back-to-back promoters could induce transcriptional interference, a phenomenon where transcriptional activity from one promoter suppresses or destabilizes the initiation of an adjacent promoter on the opposing strand.[24] Similar interference has been reported firstly in lentiviral vectors, where promoter proximity can suppress transcriptional activity by 40%–60%.[25] Given the sensitivity of the BEVS platform to process variability during industrial scale up, minor differences in physical spacing may be amplified into significant expression imbalances, resulting in higher empty capsid rates or impaired viral infectivity. Optimizing inter-promoter geometry to resolve transcriptional interference, therefore, constitutes a fundamental strategy for rational vector design.

Here, we systematically evaluated the impact of inter-promoter spacer on the coordination of Rep and Cap and the resulting rAAV quality. Using a dual-fluorescence reporter system, we mapped an optimal transcription window that enhanced transcription. This promoter-spacing optimization rebalanced the co-expression of Rep and Cap, and suppressed empty capsid formation. These findings established promoter architecture as a fundamental engineering parameter in BEVS, offering a translatable strategy to enhance both the product purity and the manufacturing efficiency of rAAV-based therapeutics.

RESULTS

Conventional back-to-back promoter architecture induces transcriptional interference

To evaluate the limitations of current BEVS-based rAAV production strategies, three distinct baculovirus configurations were compared in *Spodoptera frugiperda (Sf9)* cells. Beyond a Rep-deficient control (ITR+Cap), a separated group (ITR+Cap+Rep) was established by supplying Rep *in trans*. These were benchmarked against an integrated group (ITR+Cap-Rep), which co-expresses Rep and Cap from a conventional back-to-back *p10* and *polh* cassette (Figure 1A).

To evaluate genome replication, we quantified the relative intracellular DNA copy number of the ITR (inverted terminal repeat)-flanked *mCherry* sequence. Using qPCR on whole-cell lysates, the amplified rAAV DNA copies were normalized against the baculovirus *gp41* reference gene. Supplying Rep markedly increased replicated genome copies relative to the control, confirming that Rep is limiting for genome replication (Figure 1B). Despite encoding identical Rep and Cap proteins, the integrated configuration showed significantly lower replication than the separated system (Figure 1B). These results indicated that the close proximity of the *p10* and *polh* promoters suppressed Rep output, thereby limiting genome replication efficiency.

To elucidate the molecular basis, we profiled the transcription and translation of Rep and Cap proteins. The integrated group displayed a significant transcriptional downregulation, resulting in a severe expression imbalance (Figure 1C). Consistently, a marked loss of Rep and Cap in the integrated group relative to the separated group (Figures 1D and 1E) was observed. These data indicated that promoter proximity in the conventional bidirectional cassette suppresses Rep/Cap output, creating an unfavorable stoichiometry for genome replication.

These findings demonstrated that the conventional back-to-back *p10*/*polh* cassette in BEVS resulted in severe transcriptional interference. This architectural flaw directly suppresses Rep expression and restricts genome replication.

Inter-promoter spacer lengths of 133–161 bp enhance bidirectional promoter activity

Building on our observation that the conventional back-to-back *p10*/*polh* arrangement suppresses Rep/Cap output and compromises packaging, we reasoned that transcriptional interference might be alleviated by increasing the inter-promoter spacing. To directly confirm this idea, we established a dual-fluorescence reporter baculovirus, in which mScarlet (driven by *p10*) and EGFP (driven by *polh*) are expressed from a divergent cassette. We systematically introduced spacer sequences of varying lengths, defining the inter-promoter spacing as the interval between the transcription start sites (TSSs) of the two promoters (98–245 bp) (Figure 2A).

While we anticipated that extending the spacer would progressively mitigate interference, flow cytometry analysis revealed an unexpected non-linear response. Rather than a simple linear recovery, the screening identified a distinct "transcriptional enhancement window" between 133 and 161 bp, in which both mean fluorescence intensity (MFI) of *p10*-driven mScarlet and *polh*-driven EGFP were consistently higher than those observed with shorter or longer spacers length (Figure 2B). This transcriptional enhancement window represents an optimal inter-promoter geometry that paradoxically activates bidirectional transcription beyond what would be expected from simple spacing optimization.

Notably, mScarlet and EGFP expression remained tightly correlated across all spacer variants and time points (3–5 days post infection [dpi], $r > 0.88$), indicating that the two promoters are intrinsically

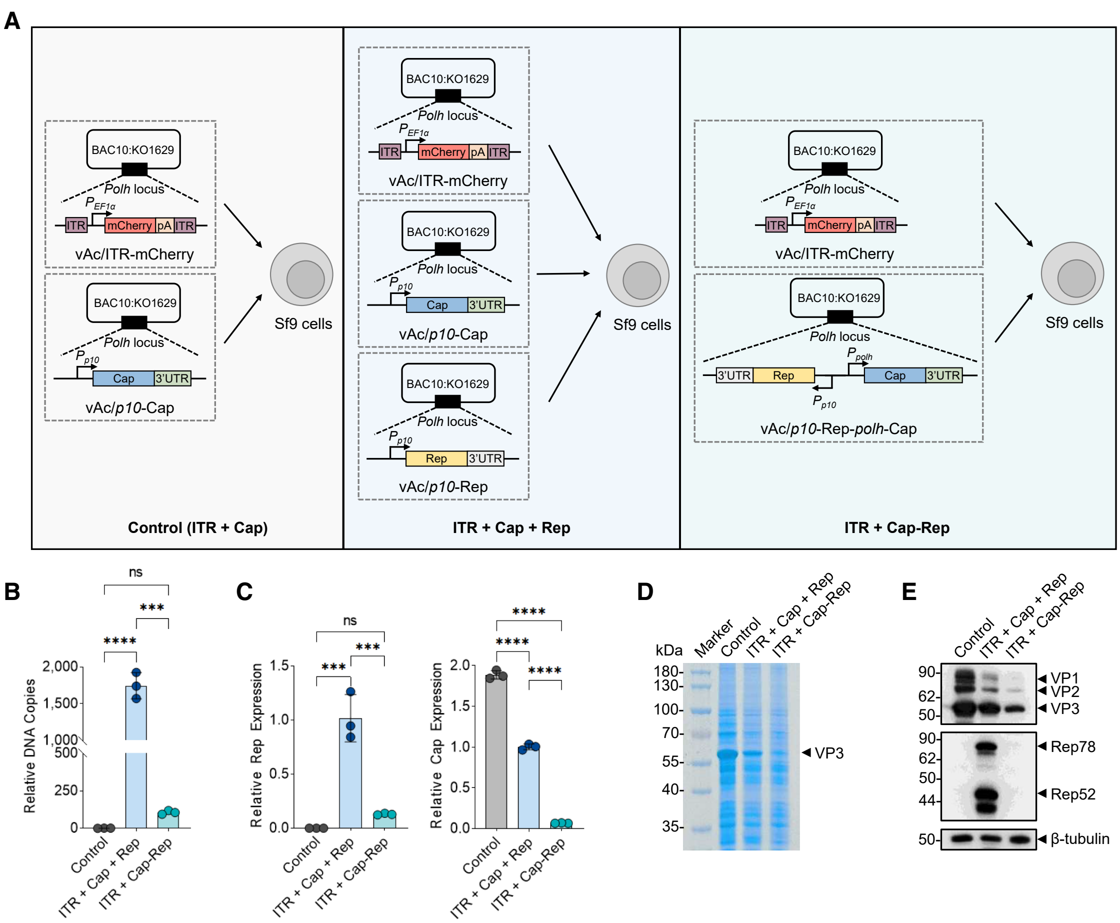

Figure 1. Conventional back-to-back bidirectional promoter architecture causes transcriptional interference

(A) Schematic diagram of three groups; *Sf*9 insect cells were co-infected with the indicated baculovirus vectors to enable transgene expression. Left: Control construct (ITR+Cap) containing Cap gene driven by the *p10* promoter (vAc/*p10*-Cap) and mCherry as a fluorescent reporter (vAc/ITR-mCherry). Middle: ITR+Cap+Rep construct with separate vAc/ITR-mCherry, vAc/*p10*-Cap, and vAc/*p10*-Rep baculoviruses, each driven by individual *EF-1α* or *p10* promoters. Right: ITR+Cap-Rep construct featuring a back-to-back bidirectional promoter cassette (vAc/*p10*-Rep-*polh*-Cap) where Rep and Cap genes are driven by *p10* and *polh* promoters. All constructs are based on BAC10:KO1629 bacmid backbone and *polh* locus integration cassettes. (B) Quantitative real-time PCR (qPCR) analysis of the relative intracellular DNA copy number of the ITR-flanked *mCherry* sequence under the three conditions shown in (A). Bars represent mean ± SD from independent biological replicates ($n = 3$). Data represent mean ± SD. One-way ANOVA with Tukey's multiple comparisons test were applied. ns, not significant; ***$p < 0.001$; ****$p < 0.0001$. (C) Quantitative analysis of relative mRNA expression for Rep (left) and Cap (right) genes. Data represent mean ± SD. One-way ANOVA with Tukey's multiple comparisons test were applied. ns, not significant; ***$p < 0.001$; ****$p < 0.0001$. (D) SDS-PAGE analysis of Cap protein expression levels from various construct. Black arrow indicates VP3 protein bands. (E) Western blotting analysis of Rep (Rep78 and Rep52) and Cap (VP1, VP2, and VP3) protein expression levels. β-tubulin serves as a loading control.

coupled within the back-to-back architecture (Figure 2C). This coordinated variation across different spacer lengths suggests that transcriptional interference operates as a system-level constraint that affects both *p10* and *polh* outputs proportionally.

These findings challenge the assumption that relieving transcriptional interference is solely dependent on increasing physical distance. Instead, they uncover a critical structural optimum (133–161 bp) that transforms the repressive back-to-back architecture into a highly active bidirectional cassette.

Identification of an optimal inter-promoter spacer window for enhanced bidirectional transcription

While the 133–161 bp window was identified as a general optimal range, we sought to map the precise structural requirements with single-nucleotide resolution. We first narrowed our focus to the 141–161 bp range, observing that both EGFP and mScarlet MFIs exhibited synchronized, non-linear fluctuations (Figure 3A). Consistent with previous observations, a robust positive correlation was maintained, confirming that spacer variations modulate the *p10*/*polh* cassette as a unified functional block (Figure 3B).

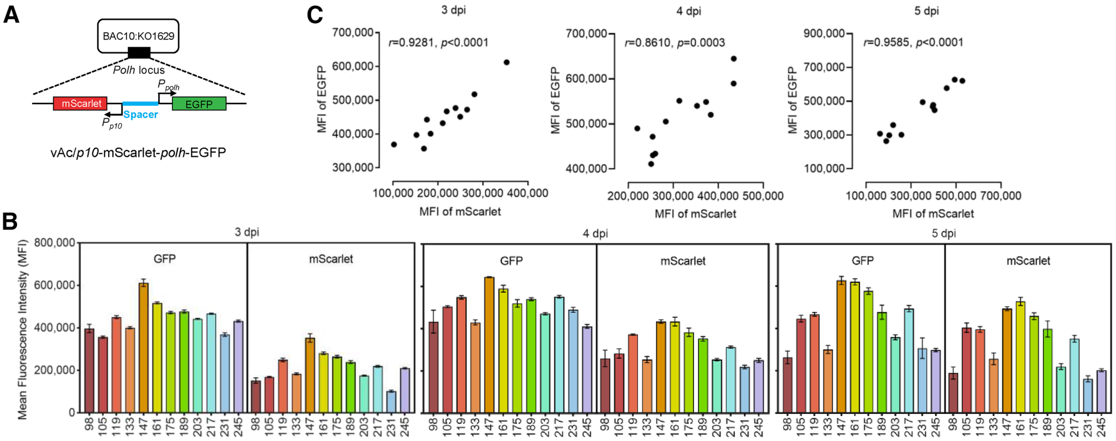

Figure 2. Dual-fluorescence reporter assay to quantify bidirectional *p10/polh* promoter activity in BEVS

(A) Schematic diagram of the dual-fluorescence reporter system. To model the back-to-back promoter architecture, a recombinant baculovirus expressing mScarlet (driven by *p10*) and EGFP (driven by *polh*) was engineered with variable spacer sequences separating the divergent promoters. (B) Quantification of reporter expression kinetics. Bar graphs display the mean fluorescence intensity (MFI) of EGFP and mScarlet measured by flow cytometry at 3, 4, and 5 days post-infection (dpi). Different colored bars represent constructs with distinct spacer lengths ranging from 98 to 245 bp. Error bars represent mean ± SD. (C) Correlation analysis of dual-promoter activity. Scatterplots depict the relationship between mScarlet and EGFP fluorescence intensities at the indicated time points. Pearson correlation coefficients (*r*) and *p* values demonstrate the expression coupling between the two reporters.

To further validate these findings and eliminate potential locus-specific effects, we established a high-resolution screening platform integrated at the *polh* locus of the BAC10:KO1629/EGFP bacmid backbone (Figure 3C). This system utilized a *p10*-mScarlet-*polh*-emiRFP670 architecture. Notably, the GFP biomarker encoded in the baculovirus backbone was employed as an internal control for normalization, accounting for variations in infection efficiency and viral replication kinetics across different constructs.

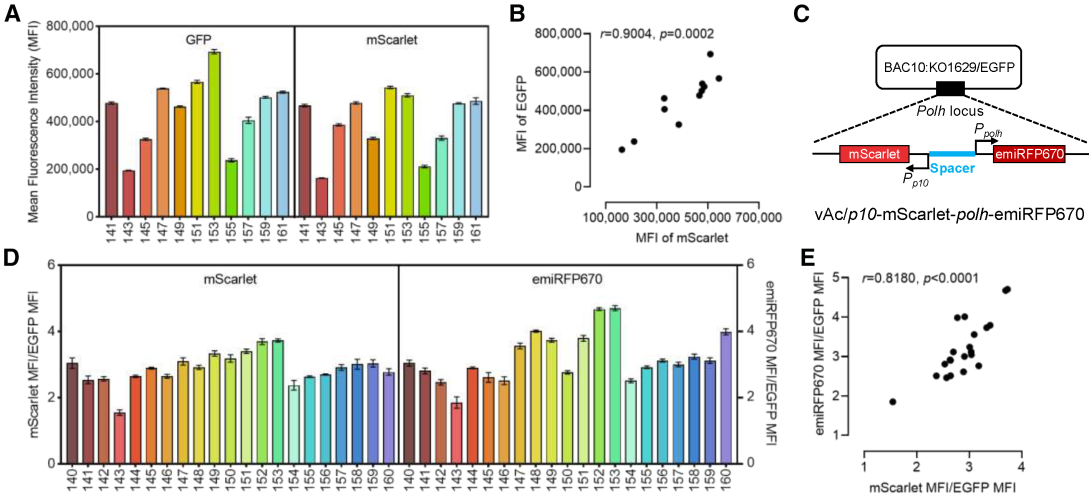

Figure 3. Identification of optimal inter-promoter spacer lengths for the bidirectional *p10/polh* architecture

(A) Quantification of reporter expression kinetics. Bar graphs display the mean fluorescence intensity (MFI) of EGFP and mScarlet measured by flow cytometry at 3 dpi. Different colored bars represent constructs with distinct spacer lengths ranging from 141 to 161 bp. Error bars represent mean ± SD. (B) Correlation analysis of dual-promoter activity. Scatterplots depict the relationship between mScarlet and EGFP fluorescence intensities at 3 dpi. Pearson correlation coefficients (*r*) and *p* values demonstrate the expression coupling between the two reporters. (C) Schematic illustration of the dual-fluorescence reporter system for high-resolution screening. To model the back-to-back promoter architecture, a recombinant baculovirus expressing mScarlet (driven by *p10*) and emiRFP670 (driven by *polh*) was engineered with variable spacer sequences lengths. All constructs were based on BAC10:KO1629/EGFP bacmid backbone and *polh* locus integration cassettes. These baculoviruses encoded a GFP biomarker to represent viral infection progress. (D) Quantification of reporter expression kinetics. Bar graphs display the MFI of mScarlet and emiRFP670 measured by flow cytometry at 3 dpi. Different colored bars represent constructs with distinct spacer lengths ranging from 140 to 160 bp. Error bars represent mean ± SD. (E) Correlation analysis of dual-promoter activity. Scatterplots depict the relationship between mScarlet and emiRFP670 fluorescence intensities at 3 dpi. Pearson correlation coefficients (*r*) and *p* values demonstrate the expression coupling between the two reporters.

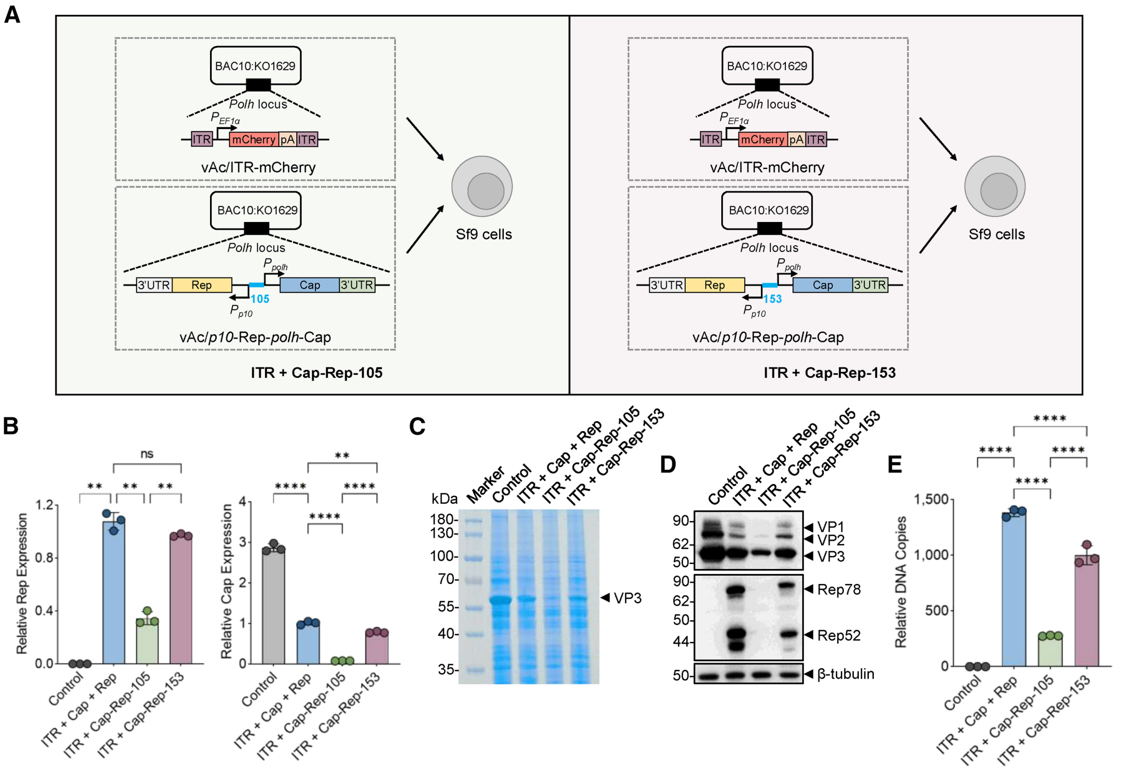

Figure 4. Optimization of the inter-promoter spacer to 153 bp maximizes bidirectional Rep/Cap output and genome replication
(A) Schematic diagram of the experimental groups comparing conventional and optimized bidirectional architectures for rAAV production. Conventional construct (ITR+Cap-Rep-105) featuring the standard back-to-back *p10*-Rep-*polh*-Cap cassette with a 105 bp spacer. Right: Optimal construct (ITR+Cap-Rep-153) utilizing the refined 153 bp spacer identified from the high-resolution screen. All constructs are integrated at the *polh* locus of the BAC10:KO1629/EGFP bacmid backbone. (B) Quantitative RT-qPCR analysis of relative mRNA expression levels for Rep (left) and Cap (right) genes. Data represent mean ± SD ($n = 3$). One-way ANOVA with Tukey's multiple comparisons test was applied. ns, not significant; $^{**}p < 0.01$; $^{****}p < 0.0001$. (C) SDS-PAGE analysis of Cap protein expression. The black arrow indicates the VP3 protein band. (D) Western blotting analysis of Rep (Rep78 and Rep52) and Cap (VP1, VP2, VP3) isoforms. β-tubulin serves as a loading control. (E) qPCR analysis of the relative intracellular DNA copy number of the ITR-flanked *mCherry* sequence across the indicated conditions. Bars represent mean ± SD ($n = 3$). Data were analyzed by one-way ANOVA with Tukey's multiple comparisons test. $^{****}p < 0.0001$.

Screening the spacer length from 140 to 160 bp in 1-bp increments confirmed the existence of distinct periodic peaks (147–153 bp) in transcriptional activity (Figure 3D). This high-resolution screening revealed that even minor adjustments in spacing could significantly impact output, yet the tight coupling between divergent reporters remained persistent (Figure 3E).

Collectively, these high-resolution findings establish that achieving peak bidirectional performance necessitates a precise structural optimum within the 147–153 bp window, effectively transforming the once-repressive back-to-back architecture into a highly active bidirectional expression platform.

The optimal 153 bp spacer configuration enhances bidirectional transcription and improves rAAV genome encapsulation

To validate the functional utility of the identified structural optimum, we integrated the refined bidirectional cassette into a rAAV packaging system. We compared our optimized 153 bp spacer (ITR+Cap-Rep-153) against the conventional 105 bp spacer (ITR+Cap-Rep-105) derived from the commercial pFastBac-Dual vector widely used in the Bac-to-Bac system (Figure 4A). The impact of spacer optimization was first identified at the transcriptional level. RT-qPCR analysis revealed that the conventional 105 bp architecture suffered from severe transcriptional repression, particularly for the Cap gene. In contrast, the 153 bp spacer effectively rescued this interference, leading to a significant increase in mRNA levels for both Rep and Cap. Notably, the optimized bidirectional system achieved Rep expression levels comparable to the separated promoter control, demonstrating full restoration of transcriptional activity (Figure 4B). This transcriptional enhancement translated directly into strong protein synthesis. SDS-polyacrylamide gel electrophoresis (SDS-PAGE) and western blotting analysis confirmed that the 105 bp construct yielded nearly undetectable levels of Rep and Cap isoforms (Figures 4C and 4D). However, the 153 bp construct exhibited high-level expression of all essential viral proteins, including Rep78, Rep52, and the capsid proteins (Figure 4D).

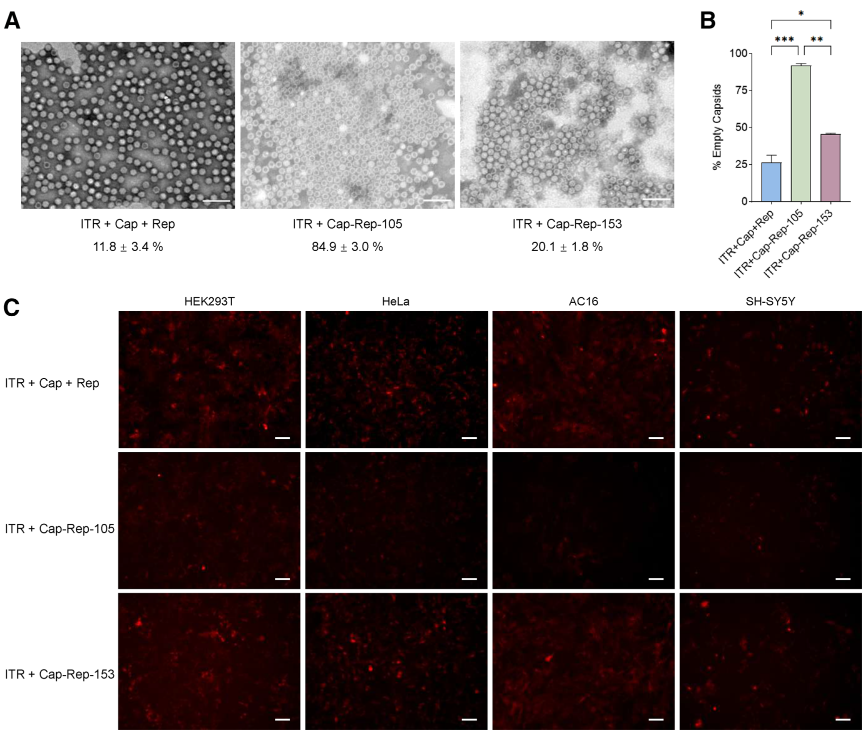

Figure 5. The optimized 153 bp inter-promoter spacer significantly improves rAAV packaging efficiency and functional transduction

(A) Transmission electron microscopy (TEM) image of rAAV particles produced with the 3-bac separated system (ITR+Cap+Rep) and 2-bac constructs (ITR+Cap-Rep-105 construct and optimized ITR+Cap-Rep-153 construct). The percentage of empty particles is indicated below (n = 2,271 particles evaluated for 153 construct, n = 3,133 for 105 construct, and n = 2,427 for 3-bac construct). Original magnification ×50,000. Scale bars, 100 nm. (B), Quantification of empty capsid ratio by the Stunner platform. Empty capsid ratios were compared among the 3-bac system (ITR+Cap+Rep), the conventional 2-bac system with a 105 bp construct (ITR+Cap+Rep-105), and the optimized 153 bp construct (ITR+Cap+Rep-153).Bars represent mean ± SD from independent biological replicates (n = 2). Data represent mean ± SD. One-way ANOVA with Tukey's multiple comparisons test were applied. *$p < 0.05$; **$p < 0.01$; ***$p < 0.001$. (C) Functional transduction of rAAV vectors. Fluorescence microscopy images of HEK293T, HeLa, AC16 (human cardiomyocytes), and SH-SY5Y (neuroblastoma) cells transduced with rAAV derived from the 3-bac, conventional 105 bp, or optimized 153 bp constructs at an MOI of 10^5 capsids/cell. Scale bars, 100 μm.

Consistent with the improved expression of packaging components, the 153 bp system demonstrated a dramatic increase in functional performance. The efficiency of rAAV genome replication, measured by the relative DNA copies of the ITR-flanked *mCherry* transgene, was approximately 3-fold higher in the 153 bp group compared with the 105 bp group (Figure 4E). To evaluate the impact of spacer optimization on final vector quality, we assessed the assembled rAAV particles using Transmission electron microscope (TEM) (Figure 5A). As anticipated, the conventional 105 bp construct yielded a high fraction of empty capsids (84.9% ± 3.0%, n = 3,133 particles). In stark contrast, the optimized 153 bp construct dramatically reduced the empty particle ratio to 20.1% ± 1.8% (n = 2,271 particles), indicating highly improved genome encapsulation. To orthogonally validate these morphological observations, we quantified the empty capsid ratios by using the Stunner platform. This absolute physical quantification corroborated the TEM data, confirming a consistent reduction in the empty capsid fraction for the 153 bp system relative to the 105 bp baseline (Figure 5B). Furthermore, we evaluated the separated three-baculovirus (3-bac) system (ITR+Cap+Rep) to serve as a functional benchmark. Both TEM (11.8% ± 3.4% empty capsids, n = 2,427 particles) and Stunner analyses confirmed that the 3-bac system yielded the highest packaging efficiency among the tested groups, supporting the premise that alleviating promoter proximity improves Rep/Cap stoichiometry.

Functionally, rAAV produced with the 153 bp spacer yielded markedly stronger mCherry expression than the 105 bp construct across HEK293T, HeLa, AC16 (human cardiomyocytes), and SH-SY5Y cells (Figure 5C). Because the transduction was performed at an equivalent capsid MOI (10^5 capsids/cell), this enhanced functional performance is directly attributable to the significantly lower empty-capsid fractions in the 153 bp and 3-bac groups.

In summary, the 153 bp spacer effectively resolves the transcriptional interference inherent to bidirectional architectures. This structural optimization ensures precise viral protein stoichiometry and superior genome encapsulation, providing a robust solution to the critical bottleneck of empty capsid contamination in rAAV manufacturing.

DISCUSSION

The manufacturing of rAAV for clinical applications requires high-volume productivity without sacrificing vector purity. While the BEVS is inherently scalable, the high frequency of empty capsids (often exceeding 80%) remains a persistent barrier. Our data indicate that this quality defect is not merely a byproduct of baculovirus biology but might be driven by the transcriptional interference inherent in conventional back-to-back promoter arrangements. The evidence is clear: when Rep is supplied *in trans* (separated system), genome replication increases markedly; however, when confined to the legacy integrated architecture, replication approaches control levels despite identical coding sequences (Figure 1B). This near-complete attenuation of Rep output (Figure 1D) directly accounts for the 84.9% ± 3.0% empty capsid rate observed by TEM (Figure 1E).

What distinguishes our findings from those of prior studies on promoter interference is the magnitude of the effect. While lentiviral studies reported a 40%–60% suppression, we observed a far more profound silencing within the bidirectional cassette. This suggests that baculovirus genomes may be particularly sensitive to promoter geometry. The tight correlation observed between *p10* and *polh* outputs (Figure 2C) indicates that these promoters do not function as independent elements but rather as an integrated functional unit responding to the same geometric constraints.

The discovery of an unexpected transcriptional enhancement window between 133 and 161 bp challenges the assumption that inter-promoter spacing serves only to alleviate steric hindrance. Rather than a simple linear recovery of activity, our dual-fluorescence screen identified a sharp functional optimum at 153 bp (Figure 2B). This non-linear response, characterized by distinct periodic peaks (Figure 3D), suggests a structural mechanism rooted in DNA topology. A spacing of 153 bp approximates the DNA length associated with a single nucleosome unit. Although baculovirus chromatin differs from that of eukaryotes, the viral DNA is packaged with basic proteins that may impose similar periodic constraints.

Translation from reporter systems to functional rAAV manufacturing confirmed the robustness of this design. By integrating the 153 bp spacer, we achieved a 3-fold increase in genome replication efficiency (Figure 4E) and reduced empty capsid accumulation to 20.1% ± 1.8% (Figure 5A). Transcriptional profiling revealed that the 153 bp configuration achieves full restoration of Rep mRNA levels, comparable to separated promoter controls (Figure 4B). Notably, the conventional 105 bp baseline exhibited a preferential suppression of the downstream promoter (Figure 4C), an asymmetry that may reflect the physical orientation of the transcription complexes within the bidirectional cluster. Our optimized system restored the coordinated expression of Rep and Cap, providing a favorable stoichiometric balance that correlates with the significant reduction in empty particle accumulation.

Rather than relying solely on steric hindrance, which would theoretically produce a progressive, asymptotic recovery curve, we hypothesize that this sharp 153 bp optimum is governed by DNA topological constraints and helical phasing. A 153 bp spacing approximates the DNA length required for a single wrap around a nucleosome-like structure. This periodicity suggests that precise angular alignment of the divergent transcription complexes, or coordinated local DNA duplex melting, may be structurally necessary for synergistic bidirectional activation. While our current findings define a functional optimum, further structural studies are required to directly interrogate these topological dynamics.

While the structural optimization of the inter-promoter spacer to 153 bp significantly rescues packaging efficiency in the integrated Rep/Cap cassette, our comparative analyses reveal that the fully separated 3-bac system still performs slightly better in minimizing empty capsids under small-scale laboratory conditions (Figures 4F and 4G). This is expected, as the physical separation of genes in the 3-bac system largely avoids transcriptional interference. However, from the perspective of industrial manufacturing, multi-vector systems introduce critical stoichiometric challenges. The efficiency of the 3-bac system is heavily reliant on the simultaneous co-infection of individual cells at precise MOI ratios—a stochastic process governed by Poisson distribution. As previously noted in BEVS scale-up studies, maintaining this consistent co-infection ratio becomes increasingly difficult to control in large-scale suspension bioreactors, frequently resulting in significant batch-to-batch variability and suboptimal product profiles.[12,26] By consolidating the Rep and Cap expression into a single cassette, the 2-bac architecture overcomes this limitation, ensuring a robust 1:1 delivery ratio to each host cell.[11] Therefore, rather than simply maximizing small-scale yield, our 153-bp spacer optimization addresses a fundamental transcriptional limitation of the industrially viable 2-bac system. This structural optimization effectively narrows the gap between scalable manufacturing and high vector purity, offering a distinct advantage over traditional optimization strategies that rely on complex bioprocess adjustments or host-cell engineering.[15,16]

Current strategies to optimize rAAV manufacturing in the BEVS largely rely on complex genetic or bioprocess modifications. Approaches to balance Rep/Cap stoichiometry include engineering stable insect cell lines,[19] consolidating genetic elements into a single baculovirus genome,[11] or tuning translation via extensive codon and intron modifications.[14] Although effective, these methods demand laborious vector reconstruction, introduce strict host-cell dependencies, and require substantial bioprocess re-validation. In contrast, we identify inter-promoter geometry as a fundamental, non-coding tuning parameter. By extending the physical spacer to 153 bp, we directly mitigate the transcriptional interference inherent to bidirectional cassettes. This spatial optimization restores viral protein stoichiometry without altering primary coding sequences, introducing new regulatory elements, or requiring specialized packaging cell lines. Consequently, this geometric design principle provides a highly modular strategy that can be readily incorporated into established BEVS platforms, improving vector quality while minimizing reconfiguration efforts.

From an industrial perspective, this geometric optimization offers a highly modular strategy to enhance manufacturing robustness. Because the improvement requires only a minor change in the DNA sequence without modifying cell lines, media, or complex bioprocess parameters. It is ideally suited for implementation in good manufacturing practice (GMP) environments where process revalidation costs are substantial. Furthermore, the architecture that maintains proportional expression between Rep and Cap may provide inherent resistance to environmental perturbations during large-scale bioreactor operations, ensuring consistent product quality across different batches.

It is important to note that this study serves as a proof-of-concept utilizing the standard AAV2 Rep/Cap and the conventional *p10*/*polh*

promoter pair. Given that varying serotypes require distinct VP1/2/3 stoichiometries, and that alternative architectures, such as the intron-containing Rep constructs described by Chen et al.,[14] introducing different regulatory dynamics, the precise 153 bp optimum may not be a universal constant across all configurations. However, the underlying principle of geometric inter-promoter tuning and the dual-fluorescence screening methodology established here provide a translatable blueprint for identifying specific spatial optima across other complex AAV production platforms.

In summary, we have shown that rational engineering of promoter architecture can effectively bypass the inherent manufacturing bottlenecks of the BEVS platform. As gene therapies move toward larger dose requirements and more complex genetic payloads, the ability to precisely calibrate transcriptional stoichiometry through structural optimization will be essential for ensuring the global accessibility and safety of high-potency genetic medicines.

METHODS

Cell culture

Sf9 insect cells (ATCC, Cat. No. CRL-1711) were cultured in SFX-Insect cell culture medium (Cytiva, Hyclone, Cat. No. SH30278.01) supplemented with 1% fetal bovine serum (FBS) at 27°C.

HEK293T, HeLa, AC16, and SH-SY5Y cells lines were obtained from Ubigene Biosciences Co., Ltd (Guangzhou, China). These cell lines were authenticated through short tandem repeat (STR) analysis and were tested negative for mycoplasma contamination. These cell lines were cultured at 37°C in a humidified atmosphere of 5% CO_2 in the following media: HEK293T, HeLa, and SH-SY5Y in DMEM (Gibco, Cat. No. C11995500BT) and AC16 in DMEM/F-12 (Gibco, Cat. No. 11320033). All culture media were supplemented with 10% FBS.

Plasmid construction

To construct the pQAV-Cap-Rep of baculovirus transfer vectors, *cap* and *rep* genes were amplified from the pAAV2/2 plasmid (Addgene, Cat. No. 232199). To construct the pQAV-*p10*-mScarlet-*polh*-EGFP-X and pQAV-*p10*-mScarlet-*polh*-emiRFP670-X series vectors, the spaces of *p10* and *polh* promoter were generated by primers (Table S1). All PCR amplifications were performed using 2 × Phanta Max Master Mix (Vazyme, Cat. No. P515). The resulting fragments were then inserted into the *p10*-mScarlet and *polh*-EGFP or *polh*-emiRFP670 using the ClonExpress Ultra One Step Cloning Kit (Vazyme, Cat. No. C116). The plasmids were verified by Sanger sequencing (Beijing Tsingke Biotech Co., Ltd).

Recombinant baculovirus production

Recombinant baculoviruses were produced using the qBac Bacmid baculovirus expression system (Bacmid Co., Ltd, China). *Sf9* cells were seeded in 6-well plates and co-transfected with linearized bacmid DNA and pQAV-X transfer plasmids using FuGENE HD Transfection Reagent (Promega, Cat. No. E2311). The transfected cells were incubated for 5 days at 27°C. Subsequently, the supernatant was collected by low-speed centrifugation (500 × *g*, 10 min), and designated as P0 recombinant baculovirus stocks.

For P1 virus amplification, *Sf9* cells were infected with P0 recombinant baculovirus stocks at an MOI of 0.05 in 100 mm cell culture dishes. Infected cells were incubated for 4 days at 27°C; the collected supernatant comprise the P1 recombinant baculovirus stocks. The P1 recombinant baculovirus stocks were subsequently titrated and utilized as the standard working stocks for all downstream analytical and preparative *Sf9* cell infections described in this study.

Western blotting

Infected cells were lysed in SDS loading buffer (50 mM Tris-HCl, 2% SDS, 10% glycerol, 1.5 mM bromophenol blue, and 100 mM DTT). The samples were denatured at 95°C for 5 min and then centrifuged at 12,000 × *g* for 1 min. Equal amounts of denatured proteins were separated by 10% SDS-PAGE. Following this, proteins were transferred to PVDF (Polyvinylidene Difluoride) membranes (Millipore, Cat. No. IPVH00010), and blocked with 5% skim milk in TBST (Tris-Buffered Saline with Tween 20) (50 mM Tris-HCl [pH 7.4], 200 mM NaCl, 0.2% Tween 20). Next, they were probed with primary antibodies overnight at 4°C, using anti-Rep monoclonal antibody (PROGEN, Cat. No. 61069; 1:300 dilution) and anti-VP1/2/3 monoclonal antibody (PROGEN, 61058; 1:300 dilution) for the detection of Rep and Cap, respectively. A horseradish peroxidase (HRP)-conjugated goat anti-mouse antibody (CoWin Biotech, Cat. No. CW0102S) was used as the secondary antibody. β-tubulin served as a loading control. Proteins were detected with Immobilon Western Chemiluminescent HRP Substrate (Millipore, Cat. No. WBKLS0100) and a ChemiDocXRS + imaging system (Bio-Rad). All uncropped scans of the blots are provided as a supplementary figure in the supplemental information (Figure S1).

Quantification of DNA copy number

To assess the replication of the ITR-flanked genome, infected cells were harvested and lysed by three cycles of freeze-thawing. The lysate was then digested with Proteinase K at a concentration of 0.2 mg/mL (Beyotime, Cat. No. ST533) for 30 min at 65°C. After digestion, the samples were denatured at 95°C for 20 min and then centrifuged at 12,000 × *g* for 15 min to pellet cellular debris. The *mCherry* gene, flanked with two ITRs, served as the target of rAAV genome. The *gp41* gene on the baculovirus genome served as the reference gene for quantification. The DNA copy number was quantified by quantitative real-time PCR using ChamQ Universal SYBR qPCR Master Mix (Vazyme, Cat. No. Q711). The relative quantity of DNA copies was calculated using the $2^{-\Delta\Delta Ct}$ method. The primer sequences were: qPCR-mCh-F: 5′ AGTTCATGTACGGCTCCAAG 3′ and qPCR-mCh-R: 5′ TTGTAGATGAACTCGCCGTC 3′; qPCR-gp41-F: 5′ CGTAGTGGTAGTAATCGCCGC 3′ and qPCR-gp41-R: 5′ AGTCGAGTCGCGTCGCTTT 3’.

rAAV production and affinity purification

Sf9 cells were seeded in 100 mm cell culture dishes and co-infected with the P1 working stocks of recombinant baculoviruses expressing

ITR, Cap, and Rep at MOI of 3 respectively. Infected cells were harvested at 5 dpi and lysed with three freeze-thaw cycles in the buffer composed of 500 mM NaCl, 2 mM $MgCl_2$ and 50 mM Tris-HCl, pH 8.0. The lysate mixture was digested with 50 U/mL benzonase (Yeasen, Cat. No. 20156ES60) for 30 min at 37°C, then centrifuged at 12,000 × g for 30 min. The clarified supernatant was collected and filtered with 0.22 μm filter. Following this, the rAAVs were purified by affinity chromatography using the POROS CaptureSelect AAVX affinity resin (Thermo Fisher Scientific, Cat. No. A36739) in accordance with the manufacturer's protocol. The purified rAAVs were detected by SDS-PAGE and concentrated using a 100 kDa Amicon filter (Millipore, Cat. No. UFC8100), followed by sterilization with 0.22 μm filter.

Transduction assay in vitro

To evaluate rAAV transduction, HEK293T, HeLa, AC16, and SH-SY5Y cells were seeded in 96-well plates at 50% confluence and transduced with purified rAAV at an MOI of 1×10^5 capsids/cell. The transgene of *mCherry* was assessed by a fluorescent microscope (DMi8, Leica, Germany) at 48 h post-transduction.

Transmission electron microscope

To evaluate particles integrity following affinity chromatography, purified rAAV samples ($\sim 10^{13}$ capsids/mL in PBS) were incubated on carbon-coated copper grids (200-mesh) for 2 min. After incubation, the grids were then negatively stained with 2% phosphotungstic acid negative stain solution (Solarbio, G1870) for 2 min. Afterward, the grids were washed with ultrapure water. Finally, the grids were air-dried and observed using TEM (HT7800, Hitachi, Japan) at a magnification of 50,000× and an accelerating voltage of 80 kV for imaging. The empty capsid ratios were determined by quantifying over 2,000 particles across four independent fields of view.

Quantification of empty capsids ratio of rAAV

Quantification of empty AAV capsids ratio was performed using the Stunner platform (Unchained Labs). Purified rAAV samples were loaded into Stunner plates, and data were acquired through combined UV/Vis spectroscopy and dynamic/static light scattering to determine the total physical viral titer and the empty/full capsid ratio according to the manufacturer's protocols.

Statistical analysis

Analyses of the statistics were conducted with GraphPad Prism 8 software. Data are presented as mean ± standard deviation of three independent replicates. The differences among the groups were analyzed by one-way analysis of variance (ANOVA) with Tukey's multiple comparisons test. Asterisks denote significance levels as follows: $^{*}p < 0.05$; $^{**}p < 0.01$; $^{***}p < 0.001$; $^{****}p < 0.0001$; and ns, not significant.

DATA AND CODE AVAILABILITY

The data that support the findings of this study are available from corresponding author upon request.

ACKNOWLEDGMENTS

We thank the Teaching and Research Core Facility at College of Life Sciences (Ningjuan Fan, Xiyan Chen, and Hui Duan), Northwest A&F University, for their support in this work. This work was supported by Chinese Universities Scientific Fund (grant no. 2452019035) in Northwest A&F University. Funding for the patent applications and related technological development was provided by Xi'an uniBac Biotechnology Co., Ltd. (K4030220184) until May 2023.

AUTHOR CONTRIBUTIONS

X.X. and H.N. conceived this project; X.X., L.S., and Y.H. supervised these experiments; X.X., H.N., and L.L. designed the research methodology; L.L. and Y.H. performed the experiments for Figure 2; L.L., Y.C., and W.M. performed the experiments for Figure 3; L.L., Y.Q., J.W., W.W., and W.S. performed the remaining experiments. L.L. and H.N. analyzed data, generated figures, and wrote the original draft; X.X., H.N., and L.L. revised the manuscript. All authors discussed the results and approved the final version of the manuscript.

DECLARATION OF INTERESTS

X.X., L.L., Y.H., and L.S. have filed patent applications (CN202380013937.X and PCT/CN2023/095761) related to this work in May 2023 through Xi'an uniBac Biotechnology Co., Ltd. and Shaanxi Bacmid Biotechnology Co., Ltd.

SUPPLEMENTAL INFORMATION

Supplemental information can be found online at https://doi.org/10.1016/j.omta.2026.201792.

REFERENCES

1. Fischer, A., and Cavazzana-Calvo, M. (2008). Gene therapy of inherited diseases. Lancet *371*, 2044–2047. https://doi.org/10.1016/S0140-6736(08)60874-0.
2. Wang, D., Tai, P.W.L., and Gao, G. (2019). Adeno-associated virus vector as a platform for gene therapy delivery. Nat. Rev. Drug Discov. *18*, 358–378. https://doi.org/10.1038/s41573-019-0012-9.
3. Muhuri, M., Levy, D.I., Schulz, M., McCarty, D., and Gao, G. (2022). Durability of transgene expression after rAAV gene therapy. Mol. Ther. *30*, 1364–1380. https://doi.org/10.1016/j.ymthe.2022.03.004.
4. Jiang, Z., and Dalby, P.A. (2023). Challenges in scaling up AAV-based gene therapy manufacturing. Trends Biotechnol. *41*, 1268–1281. https://doi.org/10.1016/j.tibtech.2023.04.002.
5. Li, C., and Samulski, R.J. (2020). Engineering adeno-associated virus vectors for gene therapy. Nat. Rev. Genet. *21*, 255–272. https://doi.org/10.1038/s41576-019-0205-4.
6. Wang, J.-H., Gessler, D.J., Zhan, W., Gallagher, T.L., and Gao, G. (2024). Adeno-associated virus as a delivery vector for gene therapy of human diseases. Signal Transduct. Targeted Ther. *9*, 78. https://doi.org/10.1038/s41392-024-01780-w.
7. Moleirinho, M.G., Silva, R.J.S., Alves, P.M., Carrondo, M.J.T., and Peixoto, C. (2020). Current challenges in biotherapeutic particles manufacturing. Expert Opin. Biol. Ther. *20*, 451–465. https://doi.org/10.1080/14712598.2020.1693541.
8. Singh, N., and Heldt, C.L. (2022). Challenges in downstream purification of gene therapy viral vectors. Curr. Opin. Chem. Eng. *35*, 100780. https://doi.org/10.1016/j.coche.2021.100780.
9. Wright, J.F. (2014). AAV empty capsids: For better or for worse? Mol. Ther. *22*, 1–2. https://doi.org/10.1038/mt.2013.268.
10. Aslanidi, G., Lamb, K., and Zolotukhin, S. (2009). An inducible system for highly efficient production of recombinant adeno-associated virus (rAAV) vectors in insect Sf9 cells. Proc. Natl. Acad. Sci. USA *106*, 5059–5064. https://doi.org/10.1073/pnas.0810614106.
11. Galibert, L., Jacob, A., Savy, A., Dickx, Y., Bonnin, D., Lecomte, C., Rivollet, L., Sanatine, P., Boutin Fontaine, M., Le Bec, C., and Merten, O.W. (2021). Monobac system–a single baculovirus for the production of rAAV. Microorganisms *9*, 1799. https://doi.org/10.3390/microorganisms9091799.
12. Joshi, P.R.H., Cervera, L., Ahmed, I., Kondratov, O., Zolotukhin, S., Schrag, J., Chahal, P.S., and Kamen, A.A. (2019). Achieving high-yield production of functional AAV5 gene delivery vectors via fedbatch in an insect cell-one baculovirus

system. Mol. Ther. Methods Clin. Dev. *13*, 279–289. https://doi.org/10.1016/j.omtm.2019.02.003.

13. Smith, R.H., Levy, J.R., and Kotin, R.M. (2009). A simplified baculovirus-AAV expression vector system coupled with one-step affinity purification yields high-titer rAAV stocks from insect cells. Mol. Ther. *17*, 1888–1896. https://doi.org/10.1038/mt.2009.128.
14. Chen, H. (2008). Intron splicing–mediated expression of AAV rep and cap genes and production of AAV vectors in insect cells. Mol. Ther. *16*, 924–930. https://doi.org/10.1038/mt.2008.35.
15. Meghrous, J., Aucoin, M.G., Jacob, D., Chahal, P.S., Arcand, N., and Kamen, A.A. (2008). Production of recombinant adeno-associated viral vectors using a baculovirus/insect cell suspension culture system: From shake flasks to a 20-L bioreactor. Biotechnol. Prog. *21*, 154–160. https://doi.org/10.1021/bp049802e.
16. Carinhas, N., Bernal, V., Yokomizo, A.Y., Carrondo, M.J.T., Oliveira, R., and Alves, P.M. (2009). Baculovirus production for gene therapy: The role of cell density, multiplicity of infection and medium exchange. Appl. Microbiol. Biotechnol. *81*, 1041–1049. https://doi.org/10.1007/s00253-008-1727-4.
17. Gray, J.T., and Zolotukhin, S. (2011). Design and Construction of Functional AAV Vectors. In Adeno-Associated Virus: Methods in Molecular Biology, R. Snyder and P. Moullier, eds. (Totowa, NJ: Humana Press), pp. 25–46. https://doi.org/10.1007/978-1-61779-370-7_2.
18. Kohlbrenner, E., Aslanidi, G., Nash, K., Shklyaev, S., Campbell-Thompson, M., Byrne, B.J., Snyder, R.O., Muzyczka, N., Warrington, K.H., and Zolotukhin, S. (2005). Successful production of pseudotyped rAAV vectors using a modified baculovirus expression system. Mol. Ther. *12*, 1217–1225. https://doi.org/10.1016/j.ymthe.2005.08.018.
19. Moreno, F., Lip, F., Rojas, H., and Anggakusuma. (2022). Development of an insect cell-based adeno-associated virus packaging cell line employing advanced rep gene expression control system. Mol. Ther. Methods Clin. Dev. *27*, 391–403. https://doi.org/10.1016/j.omtm.2022.10.015.
20. Savy, A., Kaikkonen, M.U., Léger, A., Dickx, Y., Galibert, L., and Merten, O.-W. (2018). Genetics instability of wtAAV2 genome and AAV promoter activities in the baculovirus/Sf9 cells system. PLoS One *13*, e0199866. https://doi.org/10.1371/journal.pone.0199866.
21. Chambers, A.C., Aksular, M., Graves, L.P., Irons, S.L., Possee, R.D., and King, L.A. (2018). Overview of the baculovirus expression system. Curr. Protoc. Protein Sci. *91*, 541–546. https://doi.org/10.1002/cpps.47.
22. Contreras-Gómez, A., Sánchez-Mirón, A., García-Camacho, F., Molina-Grima, E., and Chisti, Y. (2014). Protein production using the baculovirus-insect cell expression system. Biotechnol. Prog. *30*, 1–18. https://doi.org/10.1002/btpr.1842.
23. Hu, Y.-C. (2008). Baculoviral vectors for gene delivery: A review. Curr. Gene Ther. *8*, 54–65. https://doi.org/10.2174/156652308783688509.
24. Shearwin, K.E., Callen, B.P., and Egan, J.B. (2005). Transcriptional interference–a crash course. Trends Genet. *21*, 339–345. https://doi.org/10.1016/j.tig.2005.04.009.
25. Curtin, J.A., Dane, A.P., Swanson, A., Alexander, I.E., and Ginn, S.L. (2008). Bidirectional promoter interference between two widely used internal heterologous promoters in a late-generation lentiviral construct. Gene Ther. *15*, 384–390. https://doi.org/10.1038/sj.gt.3303105.
26. Mena, J.A., Aucoin, M.G., Montes, J., Chahal, P.S., and Kamen, A.A. (2010). Improving adeno-associated vector yield in high density insect cell cultures. J. Gene Med. *12*, 157–167. https://doi.org/10.1002/jgm.1420.

OMTA, Volume *34*

Supplemental information

Engineering inter-promoter spacing in baculovirus dual-expression systems enhances transcription and reduces rAAV2 empty capsids

Lan Lan, Yuchen Qu, Jie Wang, Wentao Wu, Yao Chen, Weijin Meng, Wangcheng Song, Yongqiang Hou, Lijun Shi, Hao Nan, and Xiaodong Xu

Table S1. Primers used in this study.

Primers	Sequence (5′-3′)
mSc-F	ttacttgtcatcgtcgtccttgtaatcggatcccttgtacagctcgtccatgccgccgg
mSc-R	atggtgagcaagggcgaggc
EGFP-F	atgagcaagggcgaggagct
EGFP-R	ttagtggtggtggtggtggtgctcgagcttgtacagctcgtccatgccgagagtgatcc
RFP-F	atggcggaaggctccgtcgc
RFP-R	gtggtggtggtggtggtgctcgaggctctcaagcgcggtgatccg
phRFP-R	gacggagccttccgccatatttataggttttttattacaaaactg
p10-F	cgcccttgctcaccataagcttgattgtaaataaaatgtaatttacag
polh-R	cctcgcccttgctcatgaattcatttataggttttttattacaaaactg
105-F	cgtatactccggaatattaatagatcatggagataattaaaatg
105-R	ttaatattccggagtatacggacctttaattcaacccaac
119-F	tcactcccaatagctgtcatg
119-R	atgacagctattgggagtgagacctttaattcaacccaac
133-F	caatagctgtcatggacgccatattaatagatcatggagataattaaaatgataaccat
133-R	ggcgtccatgacagctattgggagtgacagtaacggctagcgacctttaattcaacc
147-F	ttgatgactacgcagctagccgttactgtcactc
147-R	gctagctgcgtagtcatcaagacctttaattcaacccaac
161-F	ttgatgactacgcaacataaccggatacgctagccgttactgtcactc
161-R	gtatccggttatgttgcgtagtcatcaagacctttaattcaacccaac
175-F	tacgcaaccgacagacttggacataaccggatacgctagc
175-R	ccaagtctgtcggttgcgtagtcatcaagacctttaattc
189-F	accgacagacttggactgaggccagcgtacataaccggatacgctagc
189-R	acgctggcctcagtccaagtctgtcggttgcgtagtcatcaagacctttaattc
203-F	gcgtcctcttcctgaaggacataaccggatacgctagc
203-R	atgtccttcaggaagaggacgctggcctcagtccaagt
217-F	cttcctgaaggccagaatccacaccacataaccggatacgctagc
217-R	gtggattctggccttcaggaagaggacgctggcctcagtccaagt
231-F	aaggccagaatccacacccctatcctgcgcaaacataaccggatacgctagc
231-R	taggggtgtggattctggccttcaggaagaggacgctggcctcagtccaagt
245-F	gaatccacacccctatcctgcgcaatcaagctgagacaaacataaccggatacgctagc
245-R	cgcaggataggggtgtggattctggccttcaggaagaggacgctggcctcagtccaagt
141-F	aaggtcactacgcagctagccgttactgtcactc
141-R	gctagctgcgtagtgacctttaattcaacccaacac
143-F	ggtctgactacgcagctagccgttactgtcactc
143-R	gctagctgcgtagtcagacctttaattcaacccaacac
145-F	ggtcgatgactacgcagctagccgttactgtcac
145-R	tagctgcgtagtcatcgacctttaattcaacccaacac

149-F	gatgactacgcaacgctagccgttactgtcactc
149-R	gctagcgttgcgtagtcatcaagacctttaattc
151-F	tgactacgcaacatgctagccgttactgtcactc
151-R	gctagcatgttgcgtagtcatcaagacctttaattc
153-F	tgactacgcaacataagctagccgttactgtcactc
153-R	gctagcttatgttgcgtagtcatcaagacctttaattc
155-F	gactacgcaacataaccgctagccgttactgtcactc
155-R	ctagcggttatgttgcgtagtcatcaagacctttaattc
157-F	gactacgcaacataaccgggctagccgttactgtcactc
157-R	cccggttatgttgcgtagtcatcaagacctttaattc
159-F	actacgcaacataaccggatgctagccgttactgtcactc
159-R	atccggttatgttgcgtagtcatcaagacctttaattc
R140-F	ttccgcaatactattctaattcttcacatcgcattgaattcatcatggagataattaaaatgataac
R140-R	attagaatagtattgcggaataacacgagcgaagcttgacctttaattcaacccaacac
R141-F	ggttgaattaaaggtcaagcttcgctcgtgttattccgcaatactattctaattcttca
R141-R	taattatctccatgatgaattcgaatgcgatgtgaagaattagaatagtattgcgg
R142-R	taattatctccatgatgaattccgaatgcgatgtgaagaattagaatagtattgcgg
R143-R	taattatctccatgatgaattctcgaatgcgatgtgaagaattagaatagtattgcgg
R144-R	taattatctccatgatgaattcgtcgaatgcgatgtgaagaattagaatagtattgcgg
R145-R	taattatctccatgatgaattctgtcgaatgcgatgtgaagaattagaatagtattgcgg
R146-R	taattatctccatgatgaattcatgtcgaatgcgatgtgaagaattagaatagtattgcgg
R147-R	taattatctccatgatgaattcgatgtcgaatgcgatgtgaagaattagaatagtattgcgg
R148-R	taattatctccatgatgaattcagatgtcgaatgcgatgtgaagaattagaatagtattgcgg
R149-R	taattatctccatgatgaattctagatgtcgaatgcgatgtgaagaattagaatagtattgcgg
R150-R	taattatctccatgatgaattcttagatgtcgaatgcgatgtgaagaattagaatagtattgcgg
R151-R	taattatctccatgatgaattcgttagatgtcgaatgcgatgtgaagaattagaatagtattgcgg
R152-R	taattatctccatgatgaattccgttagatgtcgaatgcgatgtgaagaattagaatagtattgcgg
R153-R	taattatctccatgatgaattctcgttagatgtcgaatgcgatgtgaagaattagaatagtattgcgg
R154-R	taattatctccatgatgaattcgtcgttagatgtcgaatgcgatgtgaagaattagaatagtattgcgg
R155-R	taattatctccatgatgaattcagtcgttagatgtcgaatgcgatgtgaagaattagaatagtattgcgg
R156-R	taattatctccatgatgaattcgagtcgttagatgtcgaatgcgatgtgaagaattagaatagtattgcgg
R157-R	taattatctccatgatgaattctgagtcgttagatgtcgaatgcgatgtgaagaattagaatagtattgcgg
R158-R	taattatctccatgatgaattcatgagtcgttagatgtcgaatgcgatgtgaagaattagaatagtattgcgg
R159-R	taattatctccatgatgaattcaatgagtcgttagatgtcgaatgcgatgtgaagaattagaatagtattgcgg
R160-R	taattatctccatgatgaattcgaatgagtcgttagatgtcgaatgcgatgtgaagaattagaatagtattgcgg

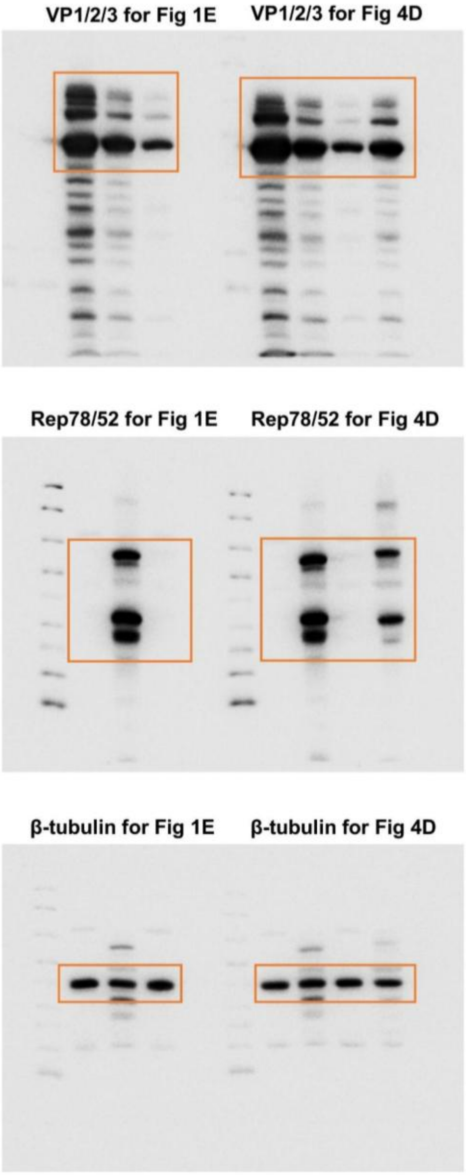

Figure S1. Uncropped scans of Western blots used in Figures 1 and 4.

Original scans of the Western blot analysis. This figure displays the original, unedited blot membranes corresponding to the cropped images presented in the main text. Top panels show the blots probed for VP1/2/3, middle panels for Rep78/52, and bottom panels for the loading control β-tubulin. The specific areas enclosed by the orange rectangles denote the cropped sections shown in Figure 1E and Figure 4D.
